# Diabetes mellitus differently affects electrical membrane properties of vagal afferent neurons of rats

**DOI:** 10.14814/phy2.15605

**Published:** 2023-02-17

**Authors:** Kerly Shamyra da Silva‐Alves, Francisco Walber Ferreira‐da‐Silva, Andrelina Noronha Coelho‐de‐Souza, Daniel Weinreich, José Henrique Leal‐Cardoso

**Affiliations:** ^1^ Laboratory of Electrophysiology, Superior Institute of Biomedical Sciences State University of Ceará Fortaleza Brazil; ^2^ Technological and Exact Science Center State University Vale do Acaraú Sobral Brazil; ^3^ Laboratory of Experimental Physiology, Superior Institute of Biomedical Sciences State University of Ceará Fortaleza Brazil; ^4^ Department of Pharmacology University of Maryland, School of Medicine Baltimore Maryland USA

**Keywords:** diabetic neuropathy, electrophysiological properties, neuronal types, nodose ganglia, sodium currents, vagal afferents

## Abstract

To study whether diabetes mellitus (DM) would cause electrophysiological alterations in nodose ganglion (NG) neurons, we used patch clamp and intracellular recording for voltage and current clamp configuration, respectively, on cell bodies of NG from rats with DM. Intracellular microelectrodes recording, according to the waveform of the first derivative of the action potential, revealed three neuronal groups (A_0_, A_inf_, and C_inf_), which were differently affected. Diabetes only depolarized the resting potential of A_0_ (from −55 to −44 mV) and C_inf_ (from −49 to −45 mV) somas. In A_inf_ neurons, diabetes increased action potential and the after‐hyperpolarization durations (from 1.9 and 18 to 2.3 and 32 ms, respectively) and reduced dV/dt_desc_ (from −63 to ‐52 V s^−1^). Diabetes reduced the action potential amplitude while increasing the after‐hyperpolarization amplitude of C_inf_ neurons (from 83 and −14 mV to 75 and −16 mV, respectively). Using whole cell patch clamp recording, we observed that diabetes produced an increase in peak amplitude of sodium current density (from −68 to −176 pA pF^−1^) and displacement of steady‐state inactivation to more negative values of transmembrane potential only in a group of neurons from diabetic animals (DB2). In the other group (DB1), diabetes did not change this parameter (−58 pA pF^−1^). This change in sodium current did not cause an increase in membrane excitability, probably explainable by the alterations in sodium current kinetics, which are also induced by diabetes. Our data demonstrate that diabetes differently affects membrane properties of different nodose neuron subpopulations, which likely have pathophysiological implications for diabetes mellitus.

## Graphical abstract

DM causes a depolarization of resting membrane potential (RMP) and decrease in action potential (AP) amplitude but not in a subpopulation of nodose neurons; Similarly, DM causes an increase in the amplitude of the total sodium current (I_Na_) and membrane conductance but not in a subpopulation of nodose neurons; Regardless of the increase in I_Na_ amplitude, DM induces no increase in neuronal excitability with was due to the displacement of steady‐state inactivation.

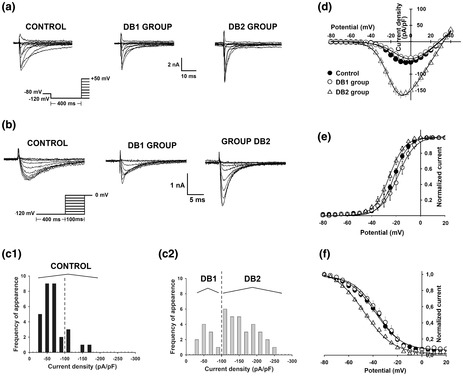

## 1 INTRODUCTION

Diabetes Mellitus (DM) is a chronic disease that affects 10.5% adult world population (536.6 million people) (Sun et al., 2022). Clinically, DM is associated with several complications that deeply compromise the quality of life and life expectancy. Among these complications, diabetic peripheral neuropathy (DPN) is the most debilitating and prevalent, affecting about 2/3 of DM patients (Garoushi et al., 2019; Pop‐Busui et al., 2017; Zhang et al., 2021). Additionally, there are many studies demonstrating that DPN can be present even in pre‐diabetic individuals (Kirthi et al., 2021).

DPN is characterized by neuronal injury accompanied by changes in excitability and action potential (AP) conduction of neurons (Krarup, 2003; Marshall et al., 2021; Quasthoff, 1998; Wada, 2006). It can injure any nerve in the body, and its signs and symptoms will vary depending on the type of neuron affected (Krarup, 2003; Quasthoff, 1998). Alterations induced by DM on somatic sensory neurons, whose somata are present in dorsal root ganglia (DRG), have been widely studied due to symptoms of paresthesia, dysesthesias, and/or neuropathic pain reported by DM patients (Pasnoor et al., 2013; Vinik et al., 2013). On the other hand, alterations induced by DM on autonomic neurons are relatively understudied (Russell & Zilliox, 2014; Vinik et al., 2013), even though these neurons are associated with serious alterations of the behavior of cardiovascular, respiratory, and gastrointestinal systems (Vinik et al., 2003; Vinik et al., 2013). Additionally, the presence of diabetic autonomic neuropathy in the patient is strongly related to the incidence of stroke and lethal arrhythmias (Cohen et al., 2003; Maser et al., 2003).

The somata of primary afferent neurons of vagus nerve, a major autonomic nerve, are located in the inferior vagal ganglia (nodose ganglion [NG]) and superior vagal ganglia (jugular ganglia) (Berthoud & Neuhuber, 2000; Zhuo et al., 1997). These neurons conduct sensory information about the state of various viscera to the central nervous system (Berthoud & Neuhuber, 2000; Zhuo et al., 1997). Vagal afferents participate in several important autonomic reflexes, such as the baroreflex, cough reflex, and vago‐vagal reflexes (Berthoud & Neuhuber, 2000; Browning & Mendelowitz, 2003). The neurons of NG, when compared to those of DRG, reveal differences in their morphology, chemosensitivity, and electrical membrane properties (Leal‐Cardoso et al., 1993; Li & Schild, 2002; Li & Schild, 2007; Undem & Weinreich, 1993; Zhuo et al., 1997), which likely gives them distinct functions.

The excitability of two distinct neuronal populations present in the NG is reduced in experimental DM, through mechanisms that differ between them. The increase in the density of HCN current (hyperpolarization‐activated cyclic nucleotide‐dependent channel) is observed in baroreceptors neurons (Li et al., 2008; Tu et al., 2010), while an increased activation of the TRESK K^+^ channel may explain the alterations observed in neurons mediating vago‐vagal reflex (Grabauskas et al., 2016). DM has been also reported to alter membrane electrophysiological properties of peripheral neurons: Somatosensory (DRG) and sympathetic ganglia (superior cervical ganglia ‐ SCG) (Djouhri et al., 2020; Ferreira‐da‐Silva et al., 2013; Hong et al., 2004; Hong & Wiley, 2006; Silva‐dos‐Santos et al., 2020). DM‐induced cellular alterations are usually attributed primarily to systemic mechanisms, such as oxidative stress, and to energy metabolism derangement (Khalid et al., 2022; Xue et al., 2021; Ye et al., 2022). DM‐induced alterations differ between neuronal populations of a single ganglion (Chen & Levine, 2001; Djouhri et al., 2020; Silva‐Alves et al., 2021; Silva‐dos‐Santos et al., 2020). It also differs from ganglion to ganglion; for example, increasing excitability in DRG and decreasing excitability in subpopulations of NG neurons (Chen & Levine, 2001; Grabauskas et al., 2016; Hong et al., 2004; Hong & Wiley, 2006; Li et al., 2008).

Based on previous studies (Chen & Levine, 2001; Djouhri et al., 2020; Silva‐Alves et al., 2021; Silva‐dos‐Santos et al., 2020), we hypothesized that in NG, DM will produce selective changes in electrophysiological membrane properties in distinct populations of NG neurons. Since different types of neurons likely have different functions; their alterations would probably have different pathophysiological implications. This would imply the necessity of elucidation of the electrophysiological alterations for a full understanding of the membrane components of DM pathophysiological mechanism. The studies of electrophysiological effect of DM on NG are restricted to one type of neuron per study and not all types were investigated, leaving a lacuna of a comprehensive investigation on DM‐induced effect on the excitability of all types of neuronal somata of NG. Thus, in order to fulfill this lacuna, in the present work we conducted a systematic investigation of the electrophysiological alterations produced by DM in different neuronal groups of NG.

## 2 MATERIALS AND METHODS

### 2.1 Animals and diabetes induction

All animal procedures were approved by the local ethic committee (process number 12777143–3 CEUA/UECE). DM was induced as described previously (Silva‐Alves et al., 2021). Briefly, 8 weeks old (170–200 g), *Wistar* rats of both sexes were randomly separated into control (CT) and diabetic (DB) groups and were fasted for 8 h. Subsequently, the DB group received a single injection of streptozotocin (STZ, 65 mg kg^−1^) diluted in sodium citrate buffer (0.1 M; pH 4.5), while the CT group was dosed with an equivalent volume of sodium citrate buffer. After 48 hours of induction, the glycemia of animals was measured and only animals with glycemia superior to 200 mg dL^−1^ were accepted into the DB group. All animals were euthanized after 4 weeks of STZ induction (12th week old).

### 2.2 Electrophysiology

#### 2.2.1 Solutions and tissues

Intact nodose ganglia were isolated and used for the intracellular recording of somal membrane electrical properties. The dissected ganglia were immediately placed in a recording chamber containing modified Locke's solution. With the aid of a stereo microscope, the connective tissue of the NG was carefully removed revealing superficial neuronal somata. Ganglia were used in the same day of dissection. The composition of the Modified Locke solution was (in mM): NaCl 140; KCl 5.6; MgCl_2_ 1.2; CaCl_2_ 2.2; tris (hydroxymethyl‐aminomethane) 10 and glucose 10, with pH adjusted to 7.4 ± 0.01.

For patch‐clamp recording, the NG was placed in Ca^2+^/Mg^2+^‐free Hank's balanced salt solutions (HBSS; pH 7.4 ± 0.01) and exposed to a short‐term dissociation protocol described by Ikeda (2004). NG was cut into small pieces with a scalpel blade using a stereo microscope. The NG pieces were transferred to a 15 mL falcon tube with a fire‐polished Pasteur pipette. The ganglia were rinsed with Ca^2+^/Mg^2+^‐free HBSS and a dissociation solution was used to obtain isolated neurons. The dissociated solution contained 1.0 mg mL^−1^ collagenase type I and 0.5 mg mL^−1^ trypsin both in HBSS. After 1 h exposure to dissociation solution, at 37°C, the falcon tube content was transferred to a 25 cm^2^ tissue culture flask that was subjected to vigorous shaking for ~10 s. The solution containing dispersed neurons was centrifuged for 5 min at 50*g*. The supernatant was removed, and the pellet was resuspended in Dulbecco's Modified Eagle's medium containing 10% fetal bovine serum, 100 U mL^−1^ streptomycin, and 0.1 mg mL^−1^ penicillin. The cells were plated on coverslips coated with poly‐D‐lysine 0.01%. The neurons were incubated in an air atmosphere containing 5% CO_2_, maintained at 37°C, and were used for current recording between 2 and 12 h after dissociation.

Ca^2+^/Mg^2+^‐free Hank's solution used in the dissociation protocol had the following composition (in mM): NaCl 137.9, KCl 5.3, KH_2_PO_4_ 0.44, NaHCO_3_ 4.0, Na_2_HPO_4_ 0.3, and glucose 5.6. For patch‐clamp recording, the composition of the bath solution was (in mM): NaCl 140.0, KCl 5.0, CaCl_2_ 1.8, MgCl_2_ 0.5, Hepes 5.0, and glucose 5.0. To study the total sodium current (I_Na_) in dissociated NG neurons we used an external solution whose composition was (in mM): NaCl 40.0, choline‐Cl 70.0, KCl 3.0, CaCl_2_ 1.0, MgCl_2_ 1.0, tetraethylammonium‐Cl 20.0, CdCl_2_ 0.1, Hepes 10.0, and glucose 10.0. The pH of all solutions was adjusted to 7.4 ± 0.01 with HCl. The pipette internal solution to measure I_Na_ contained (in mM): NaCl 10.0, CsCl 100.0, Hepes 10.0, ethylene glycol tetraacetic acid 11.0, tetraethylammonium‐Cl 10.0, MgCl_2_ 5.0, and pH adjusted to 7.2 ± 0.01 with CsOH. Choline served as the nonpermanent monovalent cation in place of external Na^+^ and was used to reduce the amplitude of the I_Na_ in patch‐clamp experiments. Cs^+^ and tetraethylammonium were used to block K^+^ channels, and Cd^2+^ was used to block Ca^2+^ channels.

#### 2.2.2 Intracellular recording of NG neurons in intact ganglia

The intact NG was fixed and transmembrane responses were recorded as described previously (Ferreira‐da‐Silva et al., 2013; Silva‐dos‐Santos et al., 2020). Briefly, the intact NG was pinned to a Silgard® polymer‐coated bottom of the recording chamber that was mounted on a magnifying glass (COLLEGE‐STEREO). The tissue was continuously perfused with modified Locke's solution through a gravitational flow system.

NG neurons were impaled with borosilicate microelectrodes fabricated on a Flaming/Brown pipette puller (P‐97, Sutter Instruments Co.) and filled with 3 M KCl solution. Resistances varied between 40 and 90 MΩ. An Ag‐AgCl wire was inserted into the microelectrode and connected to a preamplifier which, in turn, was connected to an electrometer (AXOCLAMP 900A, Molecular Devices). The Axoclamp 900A was connected to an analog/digital interface board (Digidata 1440A, Molecular Devices) which sent signals to a computer equipped with Clampex® software (p‐clamp 10, Molecular Devices) for visualization, monitoring, and signal storage for future analyzes. The microelectrode movement, as well as the impalement of the cells, was performed with an electric micromanipulator (MS 314, MW Company).

The *Current Clamp* mode was used to record the membrane electrical properties of NG neurons. For the characterization of the functional alterations of the NG neurons, three different protocols were used.

1. Protocol 1—transmembrane rectangular current pulses of −0.1 nA amplitude and 100 ms duration were used to monitor the impalement of the neurons. After impalement, the neurons were subjected to a 3–5 min rest period. A neuron was accepted for the study when it had a resting membrane potential (RMP) more negative than −40 mV.
2. Protocol 2—For the recording of the AP and passive membrane properties, rectangular pulses of 100 ms duration and amplitudes ranging from −1 to 3 nA, in 50 pA, steps were applied. With this protocol, the following parameters were quantified: RMP, input resistance (R_in_), AP threshold current (rheobase), latency, amplitude, duration, and dV/dt_asc_ and dV/dt_desc_ of the AP. Rheobase was the minimal value of stimulating current necessary to trigger AP and the latency was measured between the time of the initiation of the current stimulus and the peak of AP. The AP amplitude was measured from RMP to a maximal positive value. The duration was measured at 50% of spike amplitude. The R_in_ was measured in each cell as the ratio of small voltage drop (5–10 mV) produced by hyperpolarizing current square waves of small amplitude (−0.1 to −0.4 nA), to warrant a purely passive voltage response. The voltage drop in R_in_ measurements was done in discontinuous current clamp (DCC) and bridge mode. The measure of R_in_ was accepted correctly if the DCC measurement agreed with the bridge measurement.
3. Protocol 3—Changes in the after‐hyperpolarization (AHP) phase of the AP were quantified by the injection of rectangular current pulses of short duration (1 ms) and amplitude twice the threshold current value. The AHP duration was measured at the level of 80% recovery toward the RMP.

#### 2.2.3 Patch‐clamp recording

The coverslips with dissociated NG neurons were placed in a recording chamber on an inverted phase contrast microscope and maintained in bath solution until the recording was started. To record I_Na_ we used a perfusion system composed of storage receptacles for solutions and a perfusion pipette positioned in the vicinity (100–300 μm) of the patched cell. The rate of the perfusion solution was adjusted by gravity to ~0.3 mL min^−1^. Patch pipettes were pulled from Thick‐walled flint glass tubing (outside diameter: 1.5 mm, inside diameter: 1.1 mm, Perfecta) using a Flaming/Brown pipette puller (P‐97, Sutter Instruments). The pipettes were filled with internal solution (as described above) and had resistance ranging from 1.5 to 3.0 MΩ. Patch‐clamp recordings were made in the whole‐cell voltage‐clamp configuration using Axopatch 200B amplifier controlled by Clampex software (10.5 version, Molecular Devices). Capacitance and leakage subtraction were performed using a P/4 subtraction protocol. Series resistance compensation (70%–90%) was routinely employed to reduce voltage error. The liquid junction potential was not corrected in this set of experiments. The currents were sampled at 40 kHz and low pass filtered at 5 kHz, and data acquisition and storage were performed using computer acquisition hardware (Digidata 1440A model, Molecular Devices). All patch‐clamp recordings were made at room temperature in the range 24–26°C.

### 2.3 Na^+^ current analysis

For activation I‐V plots, total I_Na_ was evoked by the following protocol. From a holding potential of –80 mV a pre‐pulse of 400 ms duration and −120 mV in amplitude was applied to maximize sodium channels in a closed state. Voltage steps of 80 ms from −120 to +50 mV in 5 mV increments were used to elicit sodium current activation. The absolute currents elicited by voltage steps were normalized by cell capacitance to obtain the current density. The data of I‐V plots were fitted using the Ohm‐Boltzmann equation as follows:

$$I_{\mathrm{Na}+}=g_{\mathrm{Na}+\left(\max\right)}\cdot \left(V_{m}-E_{\mathrm{Na}+}\right)/\left\{1+\exp\left[-\left(V_{m}-V_{h}\right)/k\right]\right\},$$

where g_Na + (max)_ is the maximum Na^+^ conductance, V_m_ is the voltage membrane, E_Na+_ is the reversal potential for Na^+^ current, V_h_ is the voltage that activates half of the Na^+^ current and k is the slope factor.

For I_Na_ steady‐state activation curves, conductance (G) plots were constructed from Na^+^ current amplitudes by applying Ohm's law in the form: G = I/(V _m_ − E_Na+_), where V_m_ is the test potential and E_Na_ is the equilibrium potential for Na^+^ calculated using the Nernst equation. Conductances were normalized by its maximum value (G_max_) and fitted with the Boltzmann function in the form: G/G_max_ = 1/{1 + exp[(V_1/2_ − V_m_)/k]}, where V_m_ is the test potential, V_1/2_ is the half‐activation voltage and k is the slope factor. To record total I_Na_ steady‐state inactivation a 20 ms voltage step to 0 mV was initiated at the end of 80 ms depolarizing voltage pulses. The absolute currents elicited by voltage steps were normalized by membrane capacitance. For the construction of the steady‐state inactivation curve, normalized peak I_Na_ was plotted against the pre‐pulse potential, and fitted with a Boltzmann function according to the equation: I/I_max_ = 1/{1 + exp[(V_m_ – V_1/2_)/k]}, where V_m_ is the test potential, V_1/2_ is the potential at which I/I_max_ reaches its half‐maximum and k is the slope factor.

### 2.4 Statistical analysis

All results are expressed as mean ± S.E.M. The letter “*n*” indicates the number of neurons examined, and its values are shown in the corresponding figure legends or throughout the text. We used one‐way ANOVA followed by Holm‐Sidak or Bonferroni t‐test to compare the electrical properties of neuronal groups and Student t‐test to compare values between CT and DB groups. *p* < 0.05 indicates a significant statistical difference.

## 3 RESULTS

### 3.1 Characterization of the DM model

As compared to CT group, the animals dosed with STZ, named DB group (*N* = 12), developed alterations, all characteristics of DM: Hyperglycemia (425.9 mg/dL), polydipsia, polyphagia, and weight alteration (see Table 1).

**TABLE 1:** Characterization of diabetes mellitus in rats induced by streptozotocin.

Parameter	CT (*N* = 15)	DB (*N* = 12)
Glycemia (mg dL^−1^)	102.6 ± 6.47	425.9 ± 34.44*
Water intake in 24 h (mL)	33.8 ± 5.58	84.6 ± 11.72*
Food consumption in 24 h (g)	15.3 ± 1.00	24.9 ± 2.73*
Rat body weight (g)	186.6 ± 3.64	171.9 ± 5.24*

*Note*: Data are presented as mean ± S.E.M and (*N*) represents the number of animals used in each group. Abbreviations: CT, control group; DB, diabetic group.

* *p* < 0.05 when compared to the control group; one‐way ANOVA followed by Dunn's method.

### 3.2 DM‐induced alterations on different types of NG neurons

Using sharp intracellular microelectrodes, we recorded the electrical membrane properties of 65 NG neurons from CT rats and 76 neurons from NG of DB rats. The neurons of NG could be classified according to the shape of the first derivate (dV/dt) of their AP.

**FIGURE 1:**
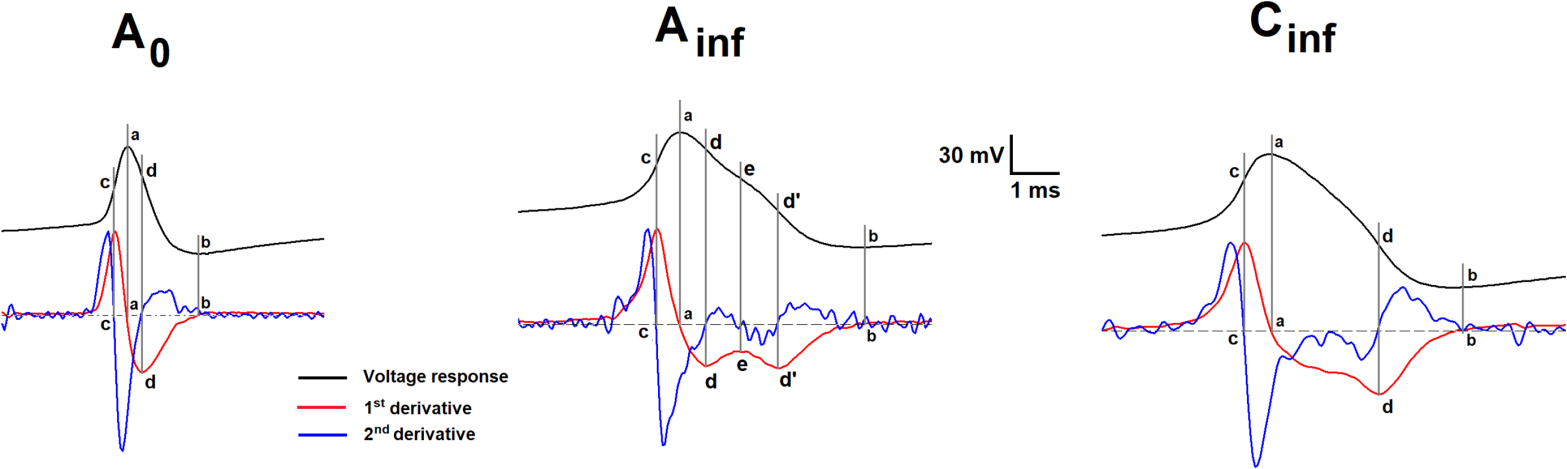
Representative traces of action potential (AP) of A_0_, A_inf_, and C_inf_ nodose ganglia neuronal groups. The top black lines represent AP's and bottom red and blue lines show 1^st^ (dV/dt) and 2^nd^ (d^2^V/dt^2^) derivatives of AP signal. Vertical gray lines crossing AP and derivatives signals represent the time at which dV/dt and d^2^V/dt^2^ are equal to zero and are also indicated by small letters for 1^st^ derivative (a and b for all neuronal groups) and 2^nd^ derivative signal (c and d for all neuronal groups and e and d' for A_inf_ neurons). The broken horizontal line represents 0.0 voltage of dV/dt signal. The calibration bar is related to the AP waveform.

**FIGURE 2:**
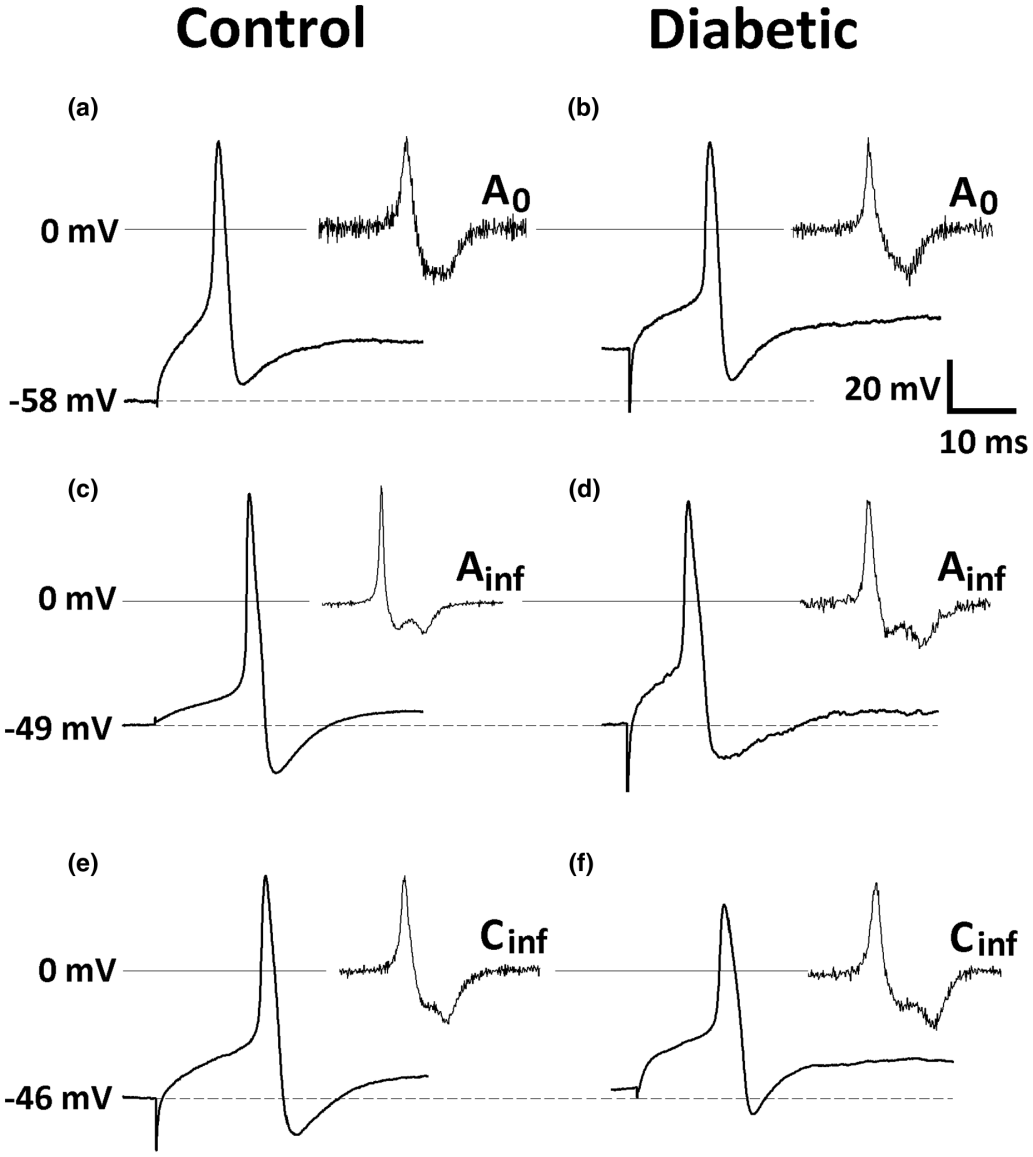
Representative traces recorded in A_0_, A_inf_, and C_inf_ neurons from nodose ganglia of control and diabetic rats. The upper traces represent A_0_ neurons, the middle traces (panels c and d) represent A_inf_ neurons, and the lower traces (panels e and f) represent C_inf_ neurons. The inserts show the first derivative of action potential that was used to classify the groups. The calibration bar is related to action potential traces.

The classification of afferent neurons using the shape of the first derivative (dV/dt) of the AP voltage, rather than the shape of AP, has the advantage of being easy to visualize and measure. Accordingly, we could discern three populations of primary afferent neurons based on the dV/dt, between the positive (vertical line *a*, Figure 1 A_0_, A_inf_, C_inf_) and the negative peak (vertical line *b*, Figure 1 A_0_, A_inf_, C_inf_) of the repolarization phase of AP: A_0_, A_inf_, and C_inf_ (see Figure 1). In A_0_ neurons, the negative values of dV/dt progress monotonically to a peak negative value (Figure 1 A_0_, Figure 2a,b), either transiently staying (Figure 2a) or not (Figure 2b) at the peak negative value, and afterward monotonically returning to 0.0 value (Figure 1 A_0_). The C_inf_ neurons show the evolution of negative values of dV/dt similar to that of A_0_, but after the transitory plateau is reached, dV/dt decreases even more reaching the peak negative value afterward (letter *d* vertical line, Figure 1 C_inf_, Figure 2e,f). In A_inf_, the dV/dt oscillates (Figure 1 A_inf_, Figure 2c,d), it is to say, it halts the decrease (vertical line *d*, Figure 1 A_inf_) to transiently increase (vertical line *e*) and then resumes the decrease, before reaching the peak negative value (vertical line *d'*). This description can be made more precise with the use of the second derivative of repolarizing phase of AP (d^2^V/dt^2^). In C_inf_ and A_inf_ neurons, during the dV/dt transient plateau (C_inf_) and the oscillation (A_inf_), the d^2^V/dt^2^ also oscillates. However, while in C_inf_, it does not surpass 0.0 value during the first wave of d^2^V/dt^2^ oscillation (see d^2^V/dt^2^ between vertical lines *a* and *d*, Figure 1 C_inf_), in A_inf_ it does so (see d^2^V/dt^2^ between vertical lines *d* and *e*, Figure 1 A_inf_). In general, C_inf_ neurons have several differences in parameter values as compared to the other types of neurons (see Figure 2 and Table 2).

**TABLE 2:** Electrophysiological parameters measured from control and diabetic nodose ganglion neurons.

Parameter	Group	A_0_	A_inf_	C_inf_
RMP (mV)	CT	−55.0 ± 4.20 (6)	−48.5 ± 1.05 (18)	−49.2 ± 1.19 (41)
	DB	−44.4 ± 1.10* (4)	−48.7 ± 1.42 (23)	−45.3 ± 1.03* (47)
Rin (MΩ)	CT	46.3 ± 18.10 (5)	66.0 ± 6.54 (11)	56.5 ± 6.24 (24)
	DB	53.4 ± 9.79 (4)	84.7 ± 10.25 (18)	51.3 ± 4.88 (35)
AHP amp. (mV)	CT	−15.0 ± 2.85 (5)	−18.1 ± 0.66^a^ (17)	−13.7 ± 0.71^b^ (37)
	DB	−24.10 ± 3.05 (4)	−15.2 ± 1.08 (25)	−15.9 ± 0.81* (45)
AHP duration_80_ (ms)	CT	31.2 ± 17.08 (5)	18.3 ± 2.02 (17)	18.1 ± 1.79 (37)
	DB	8.3 ± 1.82 (4)	32.9 ± 5.06* (25)	16.4 ± 1.52 (45)
AP amplitude (mV)	CT	72.9 ± 4.96^b^ (6)	89.6 ± 1.22^a^ ^,^ ^c^ (18)	83.4 ± 1.57^b^ (41)
	DB	52.2 ± 9.29 (4)	90.7 ± 2.36 (25)	75.0 ± 2.05* (47)
AP duration (ms)	CT	1.4 ± 0.33^a^ (6)	1.9 ± 0.09^a^ (18)	2.6 ± 0.13^b^ ^,^ ^c^ (41)
	DB	1.3 ± 0.26 (4)	2.3 ± 0.12* (25)	2.9 ± 0.25 (47)
dV/dt_asc_ (V s^−1^)	CT	125.0 ± 19.79 (6)	141.8 ± 7.48^a^ (18)	95.6 ± 5.32^b^ (41)
	DB	75.4 ± 15.52 (4)	132.4 ± 7.02 (25)	86.9 ± 6.73 (47)
dV/dt_desc_ (V s^−1^)	CT	−75.9 ± 12.34 (6)	−63.3 ± 3.12 (18)	−53.9 ± 2.35 (41)
	DB	−76.0 ± 10.40 (4)	−51.9 ± 2.61* (25)	−54.4 ± 3.50 (47)
Rheobase (pA)	CT	491.7 ± 141.08 (6)	320.6 ± 32.67^a^ (17)	519.2 ± 34.54^b^ (39)
	DB	350.0 ± 119.02 (4)	373.9 ± 43.54 (23)	580.0 ± 47.12 (45)

*Note*: Data are presented as mean ± S.E.M and (*n*) represents the number of cells.

Abbreviations: AHP, after‐hyperpolarization; AP, action potential; CT, control group; DB, diabetic group; dV/dt_asc_, maximum ascending slope of the AP; dV/dt_desc_, maximum descending slope of the AP; Rin, input resistance; RMP, resting membrane potential.

^a^ *p* < 0.05 when compared to C_inf_ neuron; one‐way ANOVA followed by Dunn's method.

^b^ *p* < 0.05 when compared to A_inf_ neuron; one‐way ANOVA followed by Dunn's method.

^c^ *p* < 0.05 when compared to A_0_ neuron; one‐way ANOVA followed by Dunn's method.

* *p* < 0.05 when compared to respective control; Student‐*t* test.

Regarding neuronal population, we observed that 9.2% (6/65), 27.7% (18/65), and 63.1% (41/65) of the NG neurons from CT rats were, respectively, A_0_, A_inf_, and C_inf_. For the NG neurons of DB rats, this distribution was, respectively 5.3% (4/76), 32.9% (25/76), and 61.8% (47/76), percentages not statistically different from respective CT values (*p* = 0.578; Chi‐square test).

**FIGURE 3:**
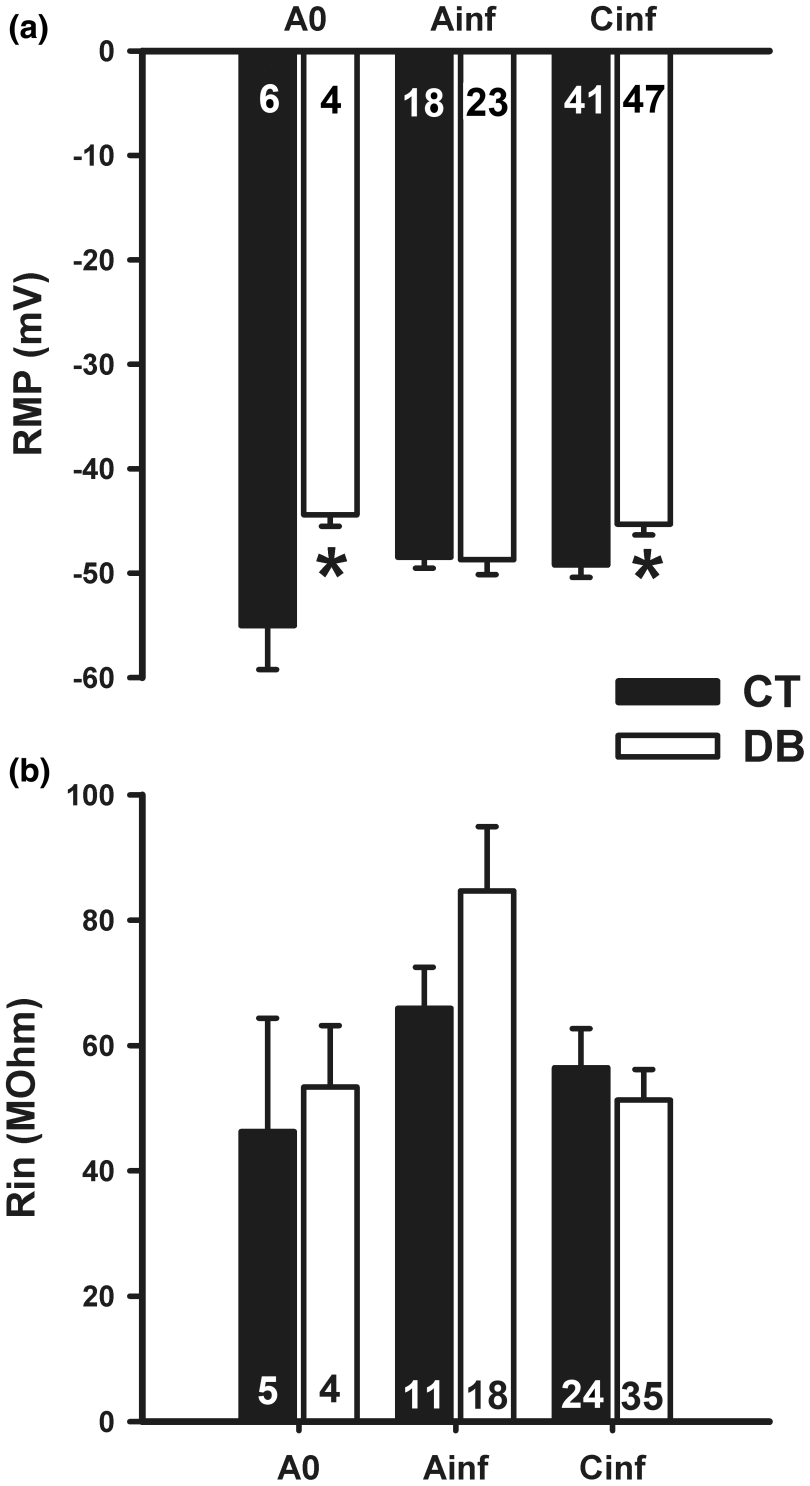
Alterations produced by diabetes mellitus on passive electrical membrane properties of nodose ganglia neuronal groups. CT, control; DB, diabetic; RMP, resting membrane potential; R_in_, input resistance; A_0_, A_inf_, and C_inf_, represent the three types of neurons, according to dV/dt of an action potential. Data are expressed as mean ± S.E.M and the number of cells is shown inside the bars. *p < 0.05, when compared to the control group (Student *t*‐test).

DM caused a statistically significant (*p* < 0.05; Student *t*‐test) depolarization of the RMP of A_0_ and C_inf_ neurons from −55.0 and −49.2 mV (CT group), respectively, to −44.4 and −45.3 mV (DB group); while did not alter the RMP of A_inf_ neurons (Figure 3a; Table 2). The R_in_ was not significantly altered by DM in each cell type (*p* > 0.05, Student *t*‐test; Figure 3b and Table 2).

**FIGURE 4:**
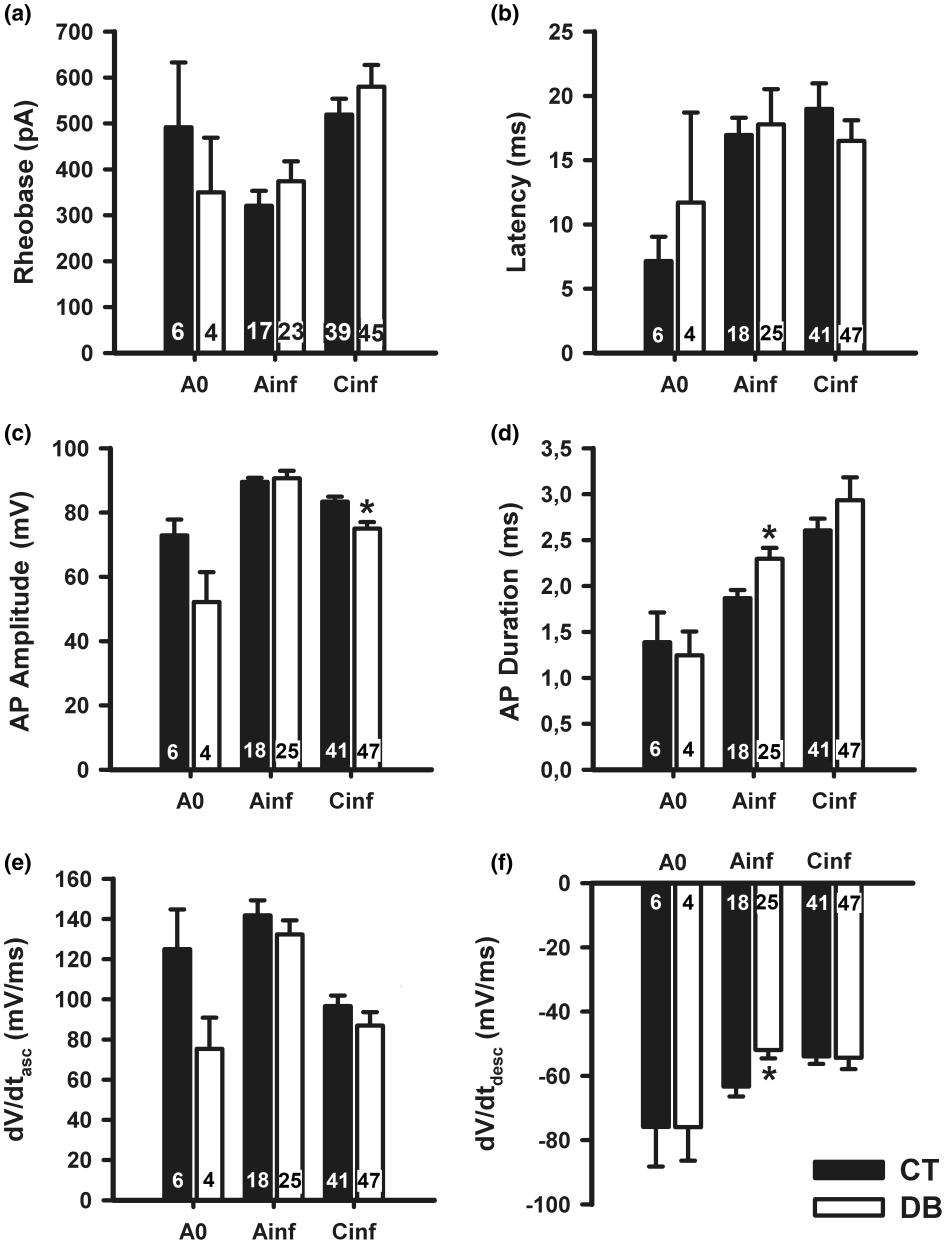
Alterations produced by diabetes mellitus on active electrical membrane properties of nodose ganglia neuronal groups. CT, control; DB, diabetic; A_0_, A_inf_, and C_inf_, the three types of neurons, were classified according to dV/dt of an action potential (AP). Data are expressed as mean ± S.E.M and the number of cells is shown inside the bars. *p < 0.05, when compared to the control group (Student *t*‐test).

DM did not significantly alter rheobase, latency, and maximum ascending slope of the AP (dV/dt_asc_) of neuronal groups studied (*p* > 0.05, Student *t*‐test; Figure 4a,b,e, and Table 2). On the contrary, DM significantly increased the AP duration and maximum descending slope of the AP (dV/dt_desc_) of A_inf_ neurons from 1.9 ms and −63.3 mV ms^−1^ to 2.3 ms and −51.9 mV ms^−1^, respectively (*p* < 0.05, Student *t*‐test; Figure 4d,f, and Table 2). Additionally, the AP amplitude of C_inf_ neurons of the DB group was significantly reduced from 89.6 mV to 75.0 mV when compared to the CT group (*p* < 0.05, Student *t*‐test; Figure 4c and Table 2).

**FIGURE 5:**
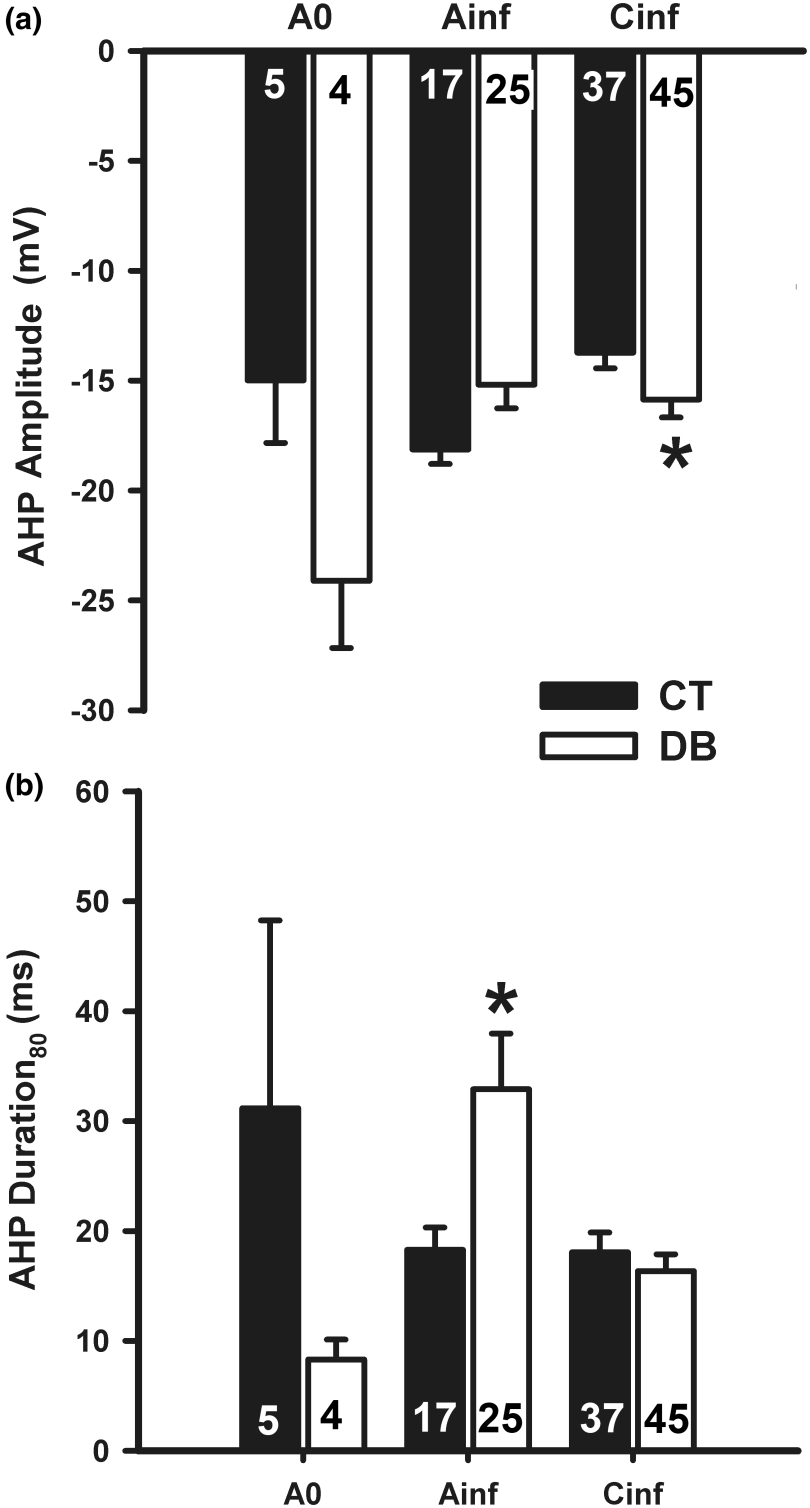
Alterations produced by diabetes mellitus on after‐hyperpolarization (AHP) parameters of nodose ganglia neuronal groups. CT, control; DB, diabetic; A_0_, A_inf_, and C_inf_, the three types of neurons, according to dV/dt of an action potential. Data are expressed as mean ± S.E.M and the number of cells is shown inside the bars. *p < 0.05, when compared to the control group (Student *t*‐test).

The amplitude and duration of the AP AHP were altered by DM. For A_inf_ neurons, the duration of AHP was increased from control 18.3 ms to 32.9 ms in the DB group, while for C_inf_ neurons the amplitude of AHP was increased from −13.7 mV to −15.9 mV (*p* < 0.05; Student *t*‐test; Figure 5 and Table 2).

### 3.3 DM‐induced alterations on total sodium currents (I_Na_) of dissociated nodose ganglia neurons

The present investigation did not find alteration of neuronal excitability in all three types of neurons (see rheobase: Figure 4 and Table 2). Since NG is a sensory ganglion similar to DRG, in which alteration does occur, we examined whether I_Na_ was altered in NG neurons.

I_Na_ of NG neurons were recorded in CT (*n* = 24) and DB (*n* = 41) groups. The mean membrane capacitance (C_m_) for all the cells were 24.6 ± 1.65 and 26.5 ± 1.25 pF, respectively, for the CT and DB groups; there were no statistically significant differences between the groups (*p* > 0.05; Student t‐test). The peak amplitude of the total I_Na_ and the I_Na_ density (I_Na_/C_m_) recorded in DB neurons (−3.6 ± 0.29 nA and 138.15 ± 9.39 pA pF^−1^, respectively) were greater than the corresponding values of the CT neurons (−1.6 ± 0.17 nA and − 64.76 ± 5.07 pA pF^−1^) (*p* < 0.001, Student t‐test).

**FIGURE 6:**
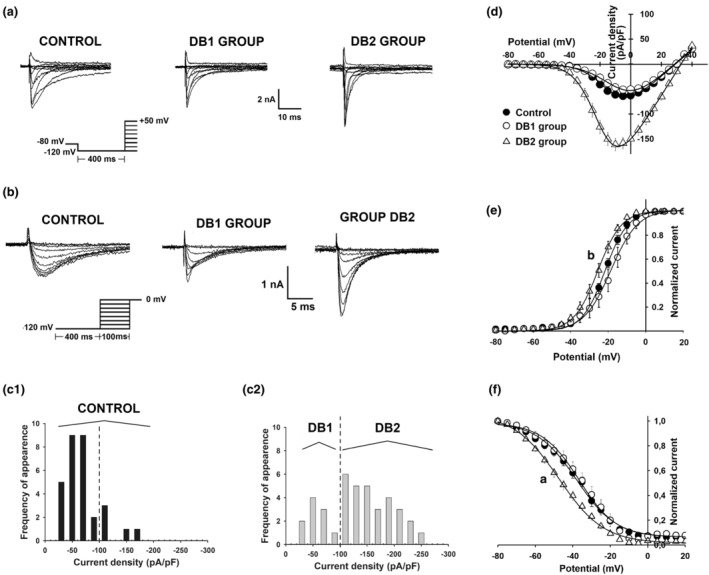
Alterations produced by diabetes mellitus on Na^+^ currents of dissociated nodose ganglion neurons. Panel a shows representative recordings of Na^+^ current activation in control (left), DB1 (center), and DB2 (right) groups. Panel b shows the Na^+^ current inactivation representative traces in the same groups. Insets in panels a and b show the voltage protocols used. Panel c is shown the frequency distribution of the peak I_Na_ of Control c1 and of Diabetic groups c2 DB1 and DB2. Panel d shows Current x Voltage relationship for I_Na_ activation. The Na^+^ current, normalized by its maximum value, at different potentials for activation and inactivation protocols, are shown in panels e and f, respectively. Small letters in panels d, e, and f indicate a statistical difference between DB2 versus control (a) and DB1 groups (b) (*p* < 0.05, ANOVA followed by Holm‐Sidak test). The number of neurons tested was 30, 10, and 31 for control, DB1 and DB2, respectively.

The visual inspection of raw data for I_Na_ and I_Na_/C_m_ peak amplitudes (data not shown) suggested that the distributions of these data in the DB group were clearly bimodal. A frequency distribution graph for current density (collected with protocols for determination of steady‐state I_Na_ activation) in CT and DB groups (Figure 6c) confirmed our hypothesis. As a result, the I_Na_ data of the DB group was segregated into two groups (Figure 6 C2) designated DB1 (*n* = 10) and DB2 (*n* = 31). Representative traces of I_Na_ current for CT, DB1, and DB2 groups are shown in Figure 6, panels A and B.

The current–voltage (I‐V) relationships for DB1 and CT groups were similar, and both differed from the I‐V relationship for the DB2 group. For transmembrane potentials positive to −40 mV, DB2 I‐V relationship showed conspicuously amplified I_Na_/C_m_ at a given transmembrane potential, as compared to CT and DB1 (Figure 6d). The peak I_Na_ density (I_Max/Cm‐(Actv)_) and the maximum conductance (G_max_) were significantly increased in the DB2 group (−175.6 pA pF^−1^ and 126.1 pS, respectively) when compared to CT (−68.4 pA pF^−1^ and 69.9 pS) and DB1 (−57.7 pA pF^−1^ and 73.8 pS) groups (Table 3). The slope (k_(actv)_) of the curves of normalized I_Na_ (Figure 6e) as a function of transmembrane potential (E_m_) in DB2 group (5.4 mV) was significantly decreased when compared to CT (7.8 mV) and DB1 (7.4 mV) groups (Table 3). The value of E_m_ at which half‐maximal steady state activation of I_Na_ occurred (V_1/2(actv)_) in DB2 (−24.9 mV) was not significantly different from V_1/2(actv)_ in CT (−21.2 mV), but it was different from DB1 (−18.5 mV) groups (table 3). The reversal potential for I_Na_ (E_Na_) in DB2 (32.6 mV) did not differ from the values of E_Na_ in CT (29.0 mV), and DB1 (29.3 mV) group (*p* < 0.05, ANOVA followed by Holm‐Sidak test).

**TABLE 3:** Electrophysiological parameters of sodium currents recorded from dissociated nodose neurons.

Parameter	CT (30)	DB1 (10)	DB2 (31)
C_m_ (pF)	24.4 ± 1.43	30.4 ± 3.76	26.5 ± 1.33
I_Max‐(Actv)_ (nA)	−1.7 ± 0.21	−1.7 ± 0.19	−4.7 ± 0.43^a^ ^,^ ^b^
I_Max/Cm‐(Actv)_ (pA pF^−1^)	−68.4 ± 6.16	−57.7 ± 5.76	−175.6 ± 12.15^a^ ^,^ ^b^
G_max_ (pS)	69.9 ± 6.43	73.8 ± 4.47	126.1 ± 8.16^a^ ^,^ ^b^
E_Na_ (mV)	29.0 ± 1.16	29.3 ± 1.83	32.6 ± 1.03
V_1/2(Actv)_ (mV)	−21.2 ± 1.06	−18.5 ± 2.75	−24.9 ± 1.42^b^
k_(Actv)_ (mV)	7.8 ± 0.31	7.4 ± 0.38	5.4 ± 0.19^a^ ^,^ ^b^
I_Max‐(Inactv)_ (nA)	−1.4 ± 0.16	−1.4 ± 0.19	−3.9 ± 0.35^a^ ^,^ ^b^
I_Max/Cm‐(Inactv)_ (pA pF^−1^)	−53.1 ± 4.54	−49.2 ± 5.67	−146.5 ± 11.48^a^ ^,^ ^b^
V_1/2(Inactv)_ (mV)	−38.9 ± 2.05	−38.5 ± 3.34	−46.6 ± 1.68^a^
k_(Inactv)_ (mV)	9.0 ± 0.73	9.8 ± 1.45	10.3 ± 0.58
A_(fast)_ (nA)	−1.2 ± 0.20	−1.0 ± 0.13	−3.4 ± 0.36^a^ ^,^ ^b^
A_(fast)_ (% of A_fast_ + A_slow_)	65.2 ± 4.21	65.7 ± 7.68	76.3 ± 2.72
τ_(fast)_ (ms)	2.3 ± 0.36	2.6 ± 0.68	1.1 ± 0.11^a^ ^,^ ^b^
A_(slow)_ (nA)	−0.5 ± 0.07	−0.6 ± 0.15	−1.1 ± 0.13^a^
A_(slow)_ (% of A_fast_ + A_slow_)	34.8 ± 4.21	38.1 ± 7.45	24.5 ± 2.69
τ_(slow)_ (ms)	13.4 ± 2.73	12.5 ± 4.12	7.5 ± 0.59

*Note*: CT; control group, DB1 and DB2; diabetic groups. Data are presented as mean ± S.E.M and (n) represents the number of cells.

Abbreviations: A_(fast)_, amplitude of I_Na_ from fast component of adjusted exponential function; A_(slow)_, amplitude of I_Na_ from slow component of adjusted exponential function; C_m_, Membrane capacitance; E_Na_, The reversal potential for I_Na_; G_max_, maximum conductance; I_Max‐(Actv)_, peak of I_Na_ on steady‐state activation; I_Max‐(Inactv)_, peak of I_Na_ on steady‐state inactivation; I_Max/Cm‐(Actv)_, density of I_Na_ on steady‐state activation; I_Max/Cm‐(Inactv)_, density of I_Na_ on steady‐state inactivation; k_(Actv)_, slope of steady‐state activation curve; k_(Inactv)_, slope of steady‐state inactivation curve; V1/2_(Actv)_, membrane potential that activates half of the current in steady‐state activation; V1/2_(Inactv)_, membrane potential that inactivates half of the current in steady‐state inactivation; τ_(fast)_, time decay from fast component of adjusted exponential function; τ_(slow)_, time decay from slow component of adjusted exponential function.

^a^ *p* < 0.05 when compared to the CT group; one‐way ANOVA followed by Dunn's method.

^b^ *p* < 0.05 when compared to the DB1 group; one‐way ANOVA followed by Dunn's method.

The I_Na_ presented exponential decay from its peak value to the asymptotic level following a fast and a slow component. The initial amplitude (A_f_) and the time constant (τ_f_) of the fast component for DB2 (−3.4 nA [76.3% of total I_Na_] and 1.1 ms, respectively) differed from A_f_ to τ_f_ of both CT (−1.2 nA [65.2% of total I_Na_] and 2.3 ms) and DB1 (−1.0 nA [65.7% of total I_Na_] and 2.6 ms) (Table 3). The initial amplitude of slow component (A_s_) for DB2 (−1.1 nA, 24.5% of total I_Na_) differed from A_s_ of the CT (−0.5 nA, 34.8% of total I_Na_) but not from the DB1 (−0.6 nA, 38.1% of total I_Na_) (Table 3). For the time constant of slow component (τ_s_), no significant difference was found between the values of DB2, CT, and DB1 (7.5, 13.4, and 12.5 ms) (Table 3).

Concerning inactivation kinetics, representative traces of I_Na_ for CT, DB1, and DB2 groups are shown in Figure 6, panel b. After the conditioning pre‐pulse at the transmembrane voltage between −120 mV and + 10 mV, the current density for the test pulse (0 mV, see insert Figure 6 for voltage clamp protocol), that was maximal (I_Max/Cm‐(Inactv)_) in all groups, in DB2 was greater and differed from both values of I_Max/Cm‐(Inactv)_ in CT and DB1 (Table 3). V_1/2(Inactiv)_ in DB2 (−46.6 mV) was significantly different from this value in CT (−38.9 mV), but not from DB1 (−38.5 mV) group (*p* < 0.05, ANOVA followed by Holm‐Sidak test) (Figure 6f; Table 3). The k_(Inactiv)_ in the DB2 group (10.3 mV) did not significantly differ from CT (9.0 mV) and DB1 (9.8 mV) (*p* > 0.05, ANOVA).

## 4 DISCUSSION

The major discovery of the present study is that experimental DM causes electrophysiological alterations in subpopulations of NG neurons. Among these alterations, there is a depolarization of RMP, an increase in the amplitude of the total I_Na_, and membrane conductance. However, these alterations were not extended to all subpopulations of nodose neurons; some subpopulations were spared. These changes caused no increase in membrane excitability, measured as the rheobase, in all subpopulations, differently from what was expected. This absence of an increase in excitability was likely because DM also displaces the curve for steady‐state inactivation of I_Na_ to more negative transmembrane potentials. To the best of our knowledge, these observations have not been previously described in the scientific literature.

The STZ‐induced DM model employed is well‐established and widely used to study DM (Akbarzadeh et al., 2007; Ferreira‐da‐Silva et al., 2013; Silva‐Alves et al., 2021; Silva‐dos‐Santos et al., 2020; Wei et al., 2003). Similarly to human DM (Akbarzadeh et al., 2007; Wei et al., 2003), the animals that received STZ developed symptomatology that mirrors the clinical disease: Hyperglycemia, polyphagia, polydipsia, and reduced gain of weight. The data obtained are in agreement with other studies with the STZ‐induced DM model (Akbarzadeh et al., 2007; Silva‐Alves et al., 2021; Silva‐dos‐Santos et al., 2020; Wei et al., 2003).

The NG is composed of different types of visceral afferent neuronal somata which are frequently classified as type A and C according to their conduction velocity, AP shape, electrophysiological properties, and chemosensitivity (Leal‐Cardoso et al., 1993; Li & Schild, 2002; Li & Schild, 2007; Undem & Weinreich, 1993; Zhuo et al., 1997). The shape of the wave of dV/dt of AP has been previously proposed for the classification of peripheral sensory ganglia neurons. This method utilizes, as the major criterion, the presence or absence of inflection in the repolarization phase of the AP (Oliveira‐Abreu et al., 2018; Oliveira‐Abreu et al., 2019; Silva‐Alves et al., 2013). The present study confirmed the presence of neurons in the NG with and without inflection in the repolarization phase of the spike of the AP.

In preliminary experiments we noted, however, a neuronal type according to the dV/dt of the repolarization phase of the AP, but also according to other parameters, did not fit either as N_0_ or N_inf_ of our previous classification (Oliveira‐Abreu et al., 2018; Oliveira‐Abreu et al., 2019; Silva‐Alves et al., 2013). Using the best criterion for classification with a visual inspection, the dV/dt of the repolarization phase of the AP, a third group of neurons was identified (A_inf_; see Figure 2, and Table 2). Since the two groups had characteristics similar to A and C neurons, we decided to call them A and C, respectively, following the literature (Harper & Lawson, 1985; Li & Schild, 2007; Oliveira‐Abreu et al., 2018; Oliveira‐Abreu et al., 2019; Silva‐Alves et al., 2013), more precisely A_0_ and C_inf_. The third group had a characteristic inflection on the dV/dt of the repolarization phase of the AP similar to C neurons. However, its electrophysiological characteristics (spike dV/dt_asc_, AP duration, AP amplitude, and rheobase) were more similar to type A neurons. Consequently, this third group was called A_inf_ (A with inflection) to distinguish it from A_0_ neurons. Li and Schild (2007) also studied neuronal types of NG correlating axonal conduction velocity with the shape and electrophysiological properties of the AP. They reported three neuronal populations: A, A_h_, and C; they also observed a neuronal type with intermediate characteristics between A and C, A_h_. Comparing the neuronal classifications made in the present study with the classification made by Li and Schild (2007), it can be inferred that neurons A_0_ and A_inf_ correspond to the somata of myelinated axons, while neurons C_inf_ correspond to the soma of unmyelinated axons. C_inf_‐type neurons were the most frequent as previously described (Leal‐Cardoso et al., 1993; Li & Schild, 2007; Undem & Weinreich, 1993).

DM produced alterations in passive electrophysiological neuronal parameters. It induced depolarization in A_0_ and C_inf_ neurons (Figure 3a). This was already expected since several studies have demonstrated that DM induces neuronal membrane depolarization (Ferreira‐da‐Silva et al., 2013; Hong et al., 2004; Silva‐Dos‐Santos et al., 2020). What was unexpected was the conspicuous absence of RMP alteration in A_inf_ neurons, in which case not even a tendency to alteration was observed.

Small, but significant alterations of AP parameters were observed in C_inf_ (decrease in AP amplitude) and A_inf_ (increase in duration and decrease in dV/dt_desc_) neurons in DM animals (Figure 4). The decrease in AP amplitude was significant in C_inf_ but only a tendency in A_0_. This is probably due to a RMP depolarization since the decrease in amplitude shows a similar magnitude as the alteration in RMP. It is noteworthy that only A_inf_ neurons underwent DM‐induced alterations of the duration and dV/dt_desc_ parameters of the AP. This fact, added to the observation that A_inf_ RMP was also not altered, reinforces the suggestion that A_inf_ is an electrophysiologic group truly different from A_0_ and C_inf_. The decrease in dV/dt_desc_ in A_inf_ is consistent with an increase in AP duration in these neurons. This increase in AP duration suggests that activation of a K^+^ conductance that controls AP repolarization (Gutman et al., 2005) is delayed in these neurons by DM.

The increase in AHP amplitude was significant in C_inf_ and was a tendency in A_0_ neurons (Figure 5a). Similar to what occurred with AP amplitude, this change in AHP amplitude is probably due to RMP depolarization. This is likely because the depolarizing alteration of RMP placed the new RMP more distant from the reversal potential of the likely K^+^ conductance (negative to the resting RMP), activated during the repolarization phase of AP (Gutman et al., 2005). If this reversal potential was not altered by DM condition, therefore, an increase in AHP amplitude was expected, as observed.

The AHP duration was significantly increased in A_inf_ neurons by DM (Figure 5b). We have no explanation for this observation. Several types of AHPs have been described that are metabolically mediated (Fowler et al., 1985; Leal‐Cardoso et al., 1993; Undem & Weinreich, 1993; Weinreich & Wonderlin, 1987). The fact that DM is predominantly a metabolic disease, suggests that metabolic, second messenger mediation of this alteration underlie changes in AHP duration.

One aspect of the DM‐induced electrophysiological alterations was the conspicuous absence of change in RMP in A_inf_ neurons. If the pathologic mechanism of DM works predominantly through a systemic mechanism, such as oxidative stress, inflammation, and increased concentration of glucose (Khalid et al., 2022; Xue et al., 2021; Ye et al., 2022), how can one understand the sparing of A_inf_ neurons? This suggests that either DM has additional pathologic mechanisms or different neuronal types have different sensitivities to the systemic physio‐pathological mechanisms of DM. Thus, in order to fully understand DM neuropathy, it is necessary to elucidate the different effects of DM on the specific types of neurons, and the specific mechanism at a given neuronal type. For example, alteration of RMP, even initially restricted to neuronal cell type itself, in a progressive disease like DM, may lead to important pathophysiological alteration. Thus, here we demonstrated that there are in DM different effects, likely relevant from the pathophysiological point of view.

This difference in the sensitivity of DM effects is perhaps due to the different expressions of ion channels in DM situation. It is known that in DRG, DM changes the expression of TTX‐S and TTX‐R channels (Hong et al., 2004; Hong & Wiley, 2006). It has also been shown that the excitability of NG baroreceptor and NG capsaicin‐sensitive neurons is reduced in diabetic animals due to changes, respectively, in HCN and K^+^ TRESK channels expression (Grabauskas et al., 2016; Li et al., 2008; Tu et al., 2010).

The absence of alteration of excitability by DM, as evaluated by measurements of rheobase (Figure 4a), was not anticipated. This was because DM increased the I_Na_ amplitude and its active membrane conductance in all DRG neurons, and this has been interpreted as an increase in excitability (Ferreira‐da‐Silva et al., 2013; Hong et al., 2004; Hong & Wiley, 2006). Due to the similarity between DRG and NG, we expected to find an increase in excitability in all NG neurons since I_Na_ is one of the major causative factors controlling the electrical excitability of all types of neurons (Catterall et al., 2005).

We determined whether DM had any effects on I_Na_ of NG neurons by voltage clamping neurons with the patch clamp technique. Surprisingly, DM, without affecting I_Na_ in a subpopulation of NG neurons designated DB1, increased I_Na_/C_m_ amplitude and its membrane conductance in another subpopulation of NG neurons, designated DB2 neurons (Figure 6).

The apparent contradictory finding of an increase in I_Na_ without an increase in excitability in any subpopulation of NG was clarified by parameters of I_Na_ in DB2 neurons. These measurements showed a large (approximately 8 mV) displacement of steady‐state I_Na_ inactivation toward more negative potentials (Figure 6). As a result of this displacement, at −50 mV RMP, the amount of Na^+^ channels available (not yet inactivated) to participate in AP generations falls from approximately 80% to 60%. Adding to this fall the repercussion of 5 mV depolarization in A_0_ and C_inf_ DB groups, the amount of available Na^+^ channels would further reduce to 50% (Figure 6). This could explain, at least partially, why the NG neurons of the DB2 subpopulation, regardless of the great increase of I_Na_, do not show an increase in excitability. Since DM did not alter I_Na_ in DB1 and DB2 it induced a displacement in I_Na_ inactivation capable of offsetting the effect of an increase in I_Na_ amplitude on excitability, the alteration of I_Na_ inactivation explains why no group of NG neurons showed an increase in excitability. It also shows a great difference in excitability alteration by DM from DRG to NG, regardless of similar effects on I_Na_ (Hong et al., 2004; Hong & Wiley, 2006).

Other studies have documented decreases in excitability in neuronal groups of NG (Grabauskas et al., 2016; Li et al., 2008; Tu et al., 2010). This differs from our results which found no excitability (as measured by rheobase) alteration. Although we have no explanation for this observation, the studies documenting a decrease in excitability worked with other functional neuronal groups whose overlap with our neuronal types is unknown. If each of their functional groups is dispersed through our types representing a small fraction in each type, the decrease in excitability may not appear as a decrease in the whole type. Alternatively, these studies used animals with 6–8 weeks of diabetes which corresponds to a longer time than the one used by us (4 weeks).

It is noteworthy the similarity of the percentage of cells not affected by DM in sharp microelectrode experiments, regarding a given parameter, as compared to the patch clamp percentage of not affect cells. This may suggest that the cells not affected by DM in patch clamp experiments would have a counterpart in cells studied with a sharp microelectrode. However, this is not the case. In the sharp microelectrodes register, all cells were somehow affected, although the affected parameter differed between groups. Because of that, with the available data, no comparison between the data collected with sharp microelectrode and patch clamp techniques is allowed.

## 5 CONCLUSION

In conclusion, we have demonstrated that DM, at the initial stage of its development, differently affected different types of NG neurons, with alterations of important parameters of cellular electrophysiology. This result implies the necessity of a knowledge of the effect of DM at the level of different neuronal populations. Comparison of NG data with those in DRG suggests important differences between NG and DRG, in DM‐induced electrophysiological alterations. Our data indicate that an extrapolation between NG to DRG (and vice versa) can be misleading.

## AUTHOR CONTRIBUTION

Kerly Shamyra da Silva‐Alves: Performed experiments, analyzed the data, and wrote the manuscript. Francisco Walber Ferreira‐da‐Silva: Performed experiments, analyzed the data, and helped to write the manuscript. Andrelina Noronha Coelho‐de‐Souza: Helped to write and revised the manuscript. Daniel Weinreich: Helped to write and revised the language and the manuscript. José Henrique Leal‐Cardoso: Conceived, revised, and helped to write the manuscript.

## FUNDING INFORMATION

This work was supported by Conselho Nacional de Desenvolvimento Científico e Tecnológico (CNPq) and Fundação Cearense de Apoio ao Desenvolvimento Científico e Tecnológico (Funcap).

## CONFLICT OF INTEREST STATEMENT

The authors declare no conflicts of interest.

## ETHICS STATEMENT

This project was approved by Animal Ethics Committee of Universidade Estadual do Ceará, process approval number 12777143‐3.
